# Dynamically orienting your own face facilitates the automatic attraction of attention

**DOI:** 10.1080/17588928.2015.1044428

**Published:** 2015-10-07

**Authors:** Minghui Liu, Xun He, Pia Rotsthein, Jie Sui

**Affiliations:** ^a^ Department of Psychology, Tsinghua University, Beijing, China; ^b^ Department of Psychology, Haerbin Normal University, Haebin, China; ^c^ Department of Psychology, Bournemouth University, Dorset, UK; ^d^ School of Psychology, University of Birmingham, Birmingham, UK; ^e^ Department of Experimental Psychology, University of Oxford, Oxford, UK

**Keywords:** Self-bias, Attentional attraction, Cueing effect, N1, P3

## Abstract

We report two experiments showing that dynamically orienting our own face facilitates the automatic attraction of attention. We had participants complete a cueing task where they had to judge the orientation of a lateralized target cued by a central face that dynamically changed its orientation. Experiment 1 showed a reliable cueing effect from both self- and friend-faces at a long stimulus onset asynchrony (SOA), however, the self-faces exclusively generated a spatial cueing effect at a short SOA. In Experiment 2, event-related potential (ERP) data to the face cues showed larger amplitudes in the N1 component for self-faces relative to friend- and unfamiliar-faces. In contrast, the amplitude of the P3 component was reduced for self compared with friend- and unfamiliar-other cues. The size of the self-bias effect in N1 correlated with the strength of self-biases in P3. The results indicate that dynamic changes in the orientation of one’s own face can provide a strong ecological cue for attention, enhancing sensory responses (N1) and reducing any subsequent uncertainty (P3) in decision-making.

Humans have an inherent ability to rapidly process a stimulus with high social significance in complex environments. For example, face images attract attention and facilitate subsequent processing at the location where they occur (Axelrod, Bar, & Rees, 2015). For example, Ro and colleagues (2001) showed that faces can act as cues to compete for spatial attention even in complex scenes (Ro et al., 2001). Furthermore, socially relevant faces can show particularly strong effects. Notably, there is evidence showing that people tend to make much faster and more accurate responses to their own faces compared to the faces of familiar others (Sui, Liu, & Han, 2009; Tong & Nakayama, 1999). Thus, researchers have reported a self-advantage effect when participants have to distinguish the orientation of face images, even though the identity of the face is irrelevant to the task (Sui & Han, 2007). Interestingly, this self-advantage effect is associated with a relatively early event-related potential (ERP) component—the anterior N2, starting around 220 ms after stimulus onset (Sui et al., 2009). However, there are still some controversial questions concerning how these effects come about. For example, do the effects occur because face identities are still coded as being task-relevant when decisions are made regarding faces (Brédart, Delchambre, & Laureys, 2006)? Does the own-face effect depend on stimuli being attended (Keyes & Dlugokencka, 2014). How early/fast does the effect occur (Tao, Zhang, Li, & Geng, 2012)? Is the effect is due to facial familiarity rather than the social significance on one’s own face (see Humphreys & Sui, in press, for a discussion of the relations between self bias and attention)? We tested here the issue of whether self-faces exert early and automatic effects on attention even when the stimuli are task irrelevant and do not have to be responded to. We used a Posner cuing paradigm, with central cues that were either the participant’s own or their friend’s face, which turned dynamically, either to the left or the right. The turning of the face was uninformative about the spatial location of a forthcoming target (cue validity was 50%), but we tested whether the cue facilitated target responses when the turning orientation was valid compared with when it was invalid. In Experiment 1, the stimulus onset asynchrony (SOA) between the cue and the target was manipulated (250 vs. 350 ms) to examine how rapidly any cueing effect from the face emerged. In Experiment 2, event-related potentials (ERPs) were recorded to the cues in the same procedure, to further test whether self-faces affect early components of responding to a stimulus and not just late stages of decision-making.

It has been debated whether self-faces act as automatic attractors of attention. Using a visual search paradigm, Tong and Nakayama (1999) reported that the self-advantage in face processing resulted from enhanced early perceptual processing due to the over-learning of familiar, own face images. In line with this, other researchers have reported that self-faces can facilitate performance even when they are processed unconsciously. For instance, using continuous flash suppression to minimize awareness for faces (self vs. other), Tao et al. (2012) found an effect of unconscious faces on a following target word which participants judged as either positive or negative in valence. The results showed that the suppressed self-faces speeded up the responses to positive words for participants with high self-esteem relative to when the words followed the suppressed face of another person. These studies fit with the idea that there is automatic processing of self-faces. However, there are also some contrary findings. For example, Ma and Han (2010) tested effects of implicit associations between the self and negative personality traits in a face-recognition task. They found that responses to implicit associations between the self and negative traits reduced the size of the self-advantage in face recognition. From this they suggested that the self-advantage effect was due to an implicit positive association between the self and positive personality traits—a high-level effect rather than an effect on early perceptual processing. Another example comes from the study of Keyes and Dlugokencka (2014). These authors tested how self-face distractors affected word naming, presenting the distractors at either central or peripheral locations. They found that responses to self and friend words were enhanced by a consistent face only when the face fell at the focus of attention. The data suggest that the self-advantage in face processing may be dependent on attentional resource being allocated to the locations where stimuli fall (also Gronau, Cohen, & Ben-Shakhar, 2003; but see Brédart et al., 2006).

In our procedure, a face cue always fell at an attended spatial location but it was spatially uninformative about the target and irrelevant to the target task (letter discrimination). We assessed whether a self-face could nevertheless cue attention to the target, and how rapidly this effect could occur (manipulating the cue-target SOA in Experiment 1 and measuring ERPs to cues, in Experiment 2).

We assumed that the dynamic self-faces would modulate early attentional responses to the cue (with a 250 ms SOA in Experiment 1), to facilitate decision-making to a following target. The latter hypothesis was examined by assessing the relations between different ERP components to the cue, in Experiment 2. Previous work has reported that the early N1 component reflects the processing of perceptual aspects of a stimulus—with a greater amplitude associated with enhanced sensory processing (Haider, Spong, & Lindsley, 1964; Luck, Woodman, & Vogel, 2000), and the late posterior P3 elicited by a cue is associated with the probability of event occurrence—in this case a small amplitude reflecting increased certainty in using cue information. For example, researchers have reported that cues with higher uncertainty in relation to a following target elicit a greater amplitude P3 (Sutton, Braren, Zubin, & John, 1965). We hypothesized that self-faces would evoke a larger amplitude N1, and a smaller amplitude P3 compared with other cues. Moreover, the size of the self-bias (vs. friend) effect in the N1 component may predict the size of the self-bias in P3. This would indicate that the degree of enhancement for sensory processing of self-faces can predict changes in the certainty of subsequent responding to the cue.

## EXPERIMENT 1: EFFECTS OF NON-INFORMATIVE DYNAMIC FACE CUES

### Method

#### Participants

Twenty college students (4 males, aged between 19 and 25 years, *M* = 22.10 ± 1.94) were paid for participation in this experiment. All were right-handed and had normal or corrected-to-normal vision. Informed consent was obtained from all participants prior to the experiment. This study was approved by a local ethics committee at Northeast Normal University of China.

#### Stimuli

**Figure 1.**
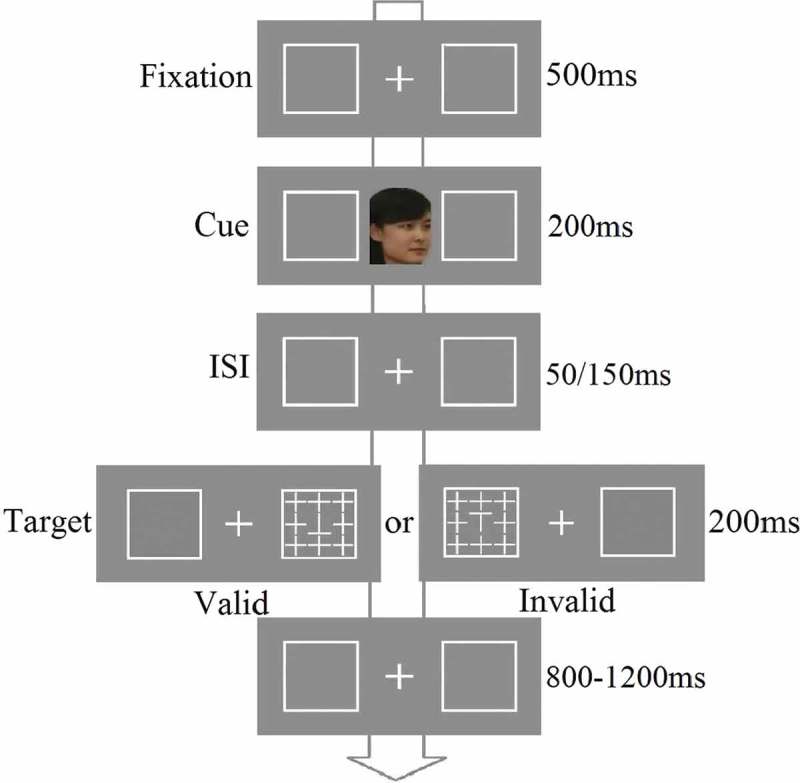
Examples of the stimuli and procedures in experiment 1. There were two types of non-informative dynamic face cue (self vs. friend).

A digital camera was used to collect 10 face images for each participant and their gender-matched best friend. The images showed five left and five right profiles of each face with a neutral facial expression, depicted at angles ranging from 15° to 75° in each direction with equal steps. When being photographed, participants were asked to keep their eye gaze straight. The images of the faces subtended about 3.2° × 4° of visual angle and they were presented in the center of the screen at a viewing distance of 60 cm. Two 3.8° × 3.8° white boxes were presented to the left and right of each face cue. The distance between the center of the display and the outer edge of each box measured 5.5° of visual angle. The target display consisted of a target letter ‘T’ embedded in the center of an array of eight cross distractors, each subtending 1.2° × 1.2° of visual angle. The letter ‘T’ was either shown upright or inverted and participants had to discriminate which was present. Figure 1 illustrates examples of the stimuli and the procedure in the experiment. All the stimuli were presented on a gray background of a 21-inch monitor (1024 × 768 at 100 Hz). E-prime 1.1 was used to control the flow of the experiment and to collect response data.

#### Procedure

Each trial began with a central fixation cross and two peripheral boxes. The fixation cross was displayed for 500 ms, then replaced by a 200 ms central face, which moved from a front orientation to turn either left or right (finishing at 90°). The dynamic movement sequence was created by showing five images at different face orientations, each for 40 ms. The SOA from the cue to the target presentation was 250 or 350 ms. After this, a target was displayed at the location where the face was looking on 50% of the trials (valid condition) or at the opposite location (the remaining 50% of the trials, the invalid condition). The next frame showed the fixation point and peripheral boxes for a variable interval ranging from 800 to 1200 ms. Participants had to judge whether the target letter ‘T’ was upright or inverted by pressing one of two keys on the keyboard (Figure 1). The instruction emphasized both response speed and accuracy. Each participant performed 288 trials after 16 practice trials. There were 36 trials per condition.

## RESULTS AND DISCUSSION

Repeated measures analyses of variance (ANOVAs) were conducted with three within-subject variables—SOA (250 ms vs. 350 ms), cue validity (valid vs. invalid), and the type of cue (self vs. friend). The analysis on accuracy performance did not show significant effects involving the type of cue, *F*s (1, 19) < 3.599, *p*s > .073, and the overall accuracy in all conditions was high (above 90%).

**Figure 2.**
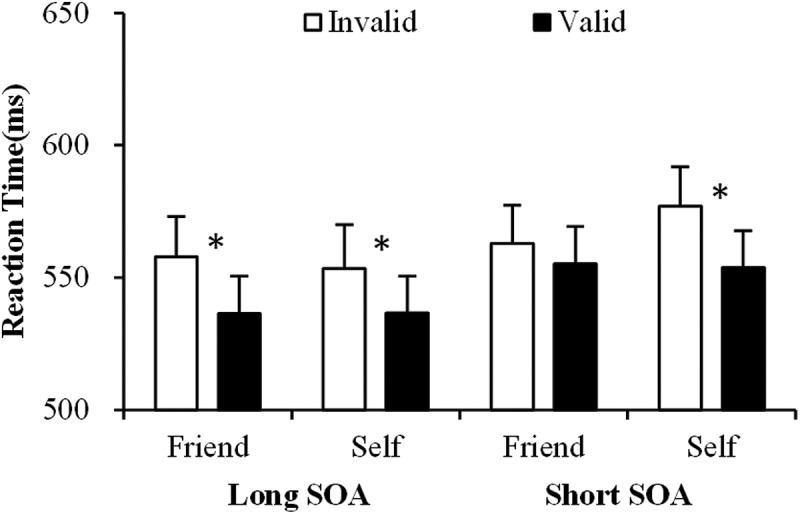
The mean reaction times (RTs) as a function of the type of cue (self vs. friend), SOA (250 vs. 350 ms), cue validity (valid vs. invalid) in experiment 1. Error bars represent standard errors.

The analyses for RTs revealed a significant main effect of SOA, *F*(1, 19) = 14.916, *p* < .005; there were slower responses in the short (250 ms) compared with the long (350 ms) SOA condition. The main effect of cue validity was also significant, *F*(1, 19) = 23.245, *p* < .001; responses to targets were faster on valid than invalid trials. These two main effects were qualified by a significant interaction between SOA and type of cue, *F*(1, 19) = 4.706, *p* < .05, but simple effect analyses failed to show any significant differences between the self- and familiar-face cues for the two SOA conditions (*p*s > .089). Importantly, there was a significant three-way interaction between SOA, cue validity and type of cue, *F*(1, 19) = 11.628, *p* < .01 (Figure 2). Follow-up analyses were conducted separately for each SOA. At the short SOA there was a significant two-way interaction between cue validity and type of cue, *F*(1, 19) = 7.580, *p* < .05. The effect was due to a larger cueing effect (valid vs. invalid) in the self cue condition (valid vs. invalid: *t*(19) = 6.439, *p* < .001) compared with the friend cue condition (valid vs. invalid: *t*(19) = 1.740, *p* = .10). In contrast, the data in the long SOA only revealed a significant main effect of cue validity, *F*(1, 19) = 12.012, *p* < .01, reflecting that the non-informative self and friend cues both guided visual attention when the SOA was long enough.

The results demonstrate that the self cue exclusively facilitated attention to a cued target in the short SOA condition. The data indicate that salient social information (the contrast between the self-face vs. a friend’s face as an attentional cue) may facilitate the automatic attraction of attention. The time course of this differential effect of the self-face on attention was tested further in Experiment 2 where we measured ERPs to cues.

## EXPERIMENT 2: AN ERP STUDY OF THE TIME COURSE OF NON-INFORMATIVE DYNAMIC FACE CUES

### Method

Fourteen paid college students (four males, aged between 21 and 26 years, *M* = 23.36 ± 1.82) participated in this experiment. The stimuli and procedure were identical to Experiment 1 expect that the SOA was changed to 600–800 ms to enable us to analyze ERPs to be measured to the cue without contamination by the response to the target. There were 16 practice trials followed by 196 experimental trials in each condition (the type of cue: Self-face, familiar friend-face, or unfamiliar stranger face). The unfamiliar other condition was included as a baseline to test the effects of face familiarity per se. Face images of one participant’s best friend were used as stranger images for other participants.

#### Electrophysiological data recording and analysis

The EEG was recorded from electrodes placed at 10–20 standard positions using a NeuroScan system (compumedicsneuroscan.com). Recordings were made with the right mastoid as a reference. All the data were re-referenced on the basis of activity on TP9 offline. The electrode impedance was kept below 5 kΩ. The EEG was amplified by using a bandpass of 0.05–30 Hz, digitized at 500 Hz. The vertical electrooculogram was monitored from two electrodes placed above and below the right eye. The horizontal electrooculogram was recorded from electrodes placed about 1.5 cm lateral to the left and right external canthi. Scan 4.5 version was used for data analyses. ERPs were averaged offline using a computer program that extracted epochs of EEG beginning 200 ms before the onset of cue and continuing for 600 ms. The ERPs of targets were not involved in data analyses because the very long SOA (600–800 ms) minimized the effects of the cue. Trials containing eye blinks, eye movement deflections exceeding ±50 μV at any electrode, or incorrect behavioral responses were excluded from the ERP averages. There were 12.5% trials in total excluded from data analyses. The baseline for ERP measurements was the mean voltage of a 200-ms prestimulus interval and the latency was measured relative to the onset of the cue.

The grand-average ERPs to the self, familiar, and unfamiliar face cues were characterized by N1—a negativity waveform around 150–190 ms over the central-parietal area. The three types of cue mainly differed in activity over the N1 time period over the left central-parietal region, hence, the brain activity in FT7, T7, C5, C3, C1, CP3, and CP1 electrodes was taken to conduct the statistical analysis. There was also a face-specific component—an N170 in the bilateral temporal-occipital region elicited by the dynamic face cues. To assess this, data from six electrodes (PO7, PO5, P7, P5, PO8, PO6, P8, and P6) were extracted for the following analyses. In addition, a late positive component P3 in a 400–600 ms time window (with a peak around 546 ms) was evident over the frontal, central, and parietal areas. The difference in this component across the three types of cues occurred over the right hemisphere; accordingly, data from eight electrodes (F8, FC6, C4, C6, T8, CP2, CP4, and TP8) were extracted for the analyses.

## RESULTS AND DISCUSSION

### Behavioral data

Repeated measures analyses of variance (ANOVAs) with two within-subjects variables—the type of cue (self, friend, or unfamiliar other) and cue validity (valid vs. invalid)—were conducted for accuracy and RTs respectively. The mean accuracy was above 89%. The ANOVAs for accuracy performance and RTs failed to show any significant effects involving cue type, *F*s < 1, *p*s > .05. There was only a significant main effect of cue validity in RTs, *F*(1, 13) = 5.588, *p* = .034; there were faster responses to the valid than invalid cue conditions. The lack of differential effects of cue type here likely reflects the long SOA used between the cue and the target.

### Electrophysiological data on responses to cues

We conducted ANOVAs with one within-subject variable—the type of cue (self, friend, or unfamiliar)—for each electrode for the three components of interest: N1, N170, and P3 respectively.

**Figure 3.**
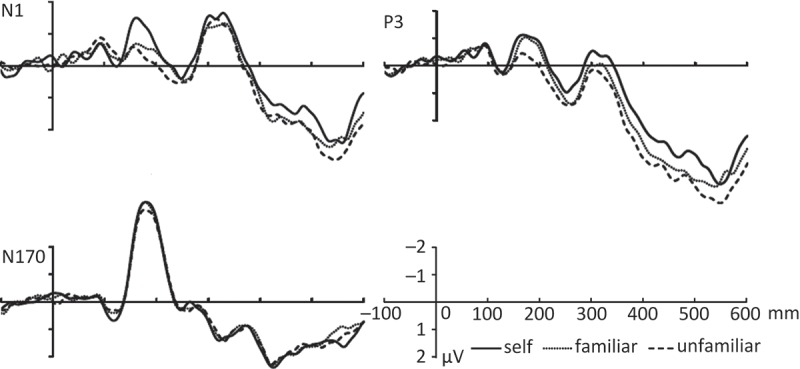
The mean ERPs in the N1, P3, and N170 over the selective electrodes as a function of the type of cue (self, friend, or unfamiliar other) in experiment 2.

*N1*—The analysis revealed a significant effect of the type of cue across the left central-parietal region (FT7, T7, C5, C3, C1, CP3, and CP1), *F*(2, 26) = 4.275–10.419, *p* < .05; post-doc pairwise comparisons revealed that the self cue elicited larger amplitudes over the left central-parietal region than friend and unfamiliar other cues, (*p* < .035), but there was no difference between the friend and unfamiliar cues (*p* > .05) (Figure 3A). The data indicate that the social saliency of self-relevant cues facilitated early attentional responses to the face cues. Given the lack of difference between the friend and stranger conditions, this effect seems unlikely to be driven in any simple linear way by the familiarity of the stimulus.

*N170*—There was no significant effect of cue for the N170 over the average of the PO7, PO5, P7, P5, PO8, PO6, P8, and P6 electrodes, *F*(2, 26) = 1.558, *p* = .23 (Figure 3B).

*P3*—The analysis showed a significant effect of cue over the right frontal-central regions including electrode locations F8, FC6, C4, C6, T8, CP2, CP4, and TP8, *F*(2, 26) = 3.780–11.551, *p* < .05. Post-doc pairwise comparisons indicated that there were smaller amplitudes in the self than in the friend and unfamiliar cue conditions over the average of the electrodes (*p* < .033); there was no difference between the friend and the unfamiliar face cues (*p*s > .05) (Figure 3C).

*Correlation analyses*—To test the hypothesis that the early perceptual sensory driven component (the effect of self on the N1) can predict the activity in cue-related decision-making, indexed by the P3 component, correlation analyses were performed across participants for the measures of self-bias and friend-bias. Self-bias was defined by the differential scores between the amplitudes of each component following the self and friend cues (friend minus self). Friend-bias was indexed by the differential scores between the amplitude of each component following the friend and unfamiliar other cues (unfamiliar minus friend). The analysis revealed a significant positive correlation between the N1 and P3 components in the self-bias effect, *r* = .723, *p* < .005, indicating that the size of the self-bias in N1 predicted the magnitude of self-bias in P3, suggesting that the degree of perceptual enhancement for self-related stimuli was related to the reduced uncertainty of decision-making reflected in the P3. In contrast, there was no significant correlation between the N1 and P3 amplitudes for the friend-bias effect, *r* = .394, *p* = .164. The null result with the friend stimuli indicates that the relationship between the biases in the N1 and P3 components was unlikely to be driven by the familiarity of self-related stimuli.

No significant correlations were observed in self-bias and friend-bias between the amplitudes of the N170 and P3 components, *r* < .360, *p* > .204.

The data indicate that there was enhancement of an early ERP component (the N1) for self-face cues compared to other face cues (friend and stranger), consistent with an effect of the self on the early orienting of attention. In addition, the magnitude of the enhancement for the N1 component correlated with reductions in the size of the P3 component. This result fits with the idea that a stronger early attentional response to the cue reduced subsequent uncertainty about using the cue for orienting to the target.

## GENERAL DISCUSSION

In the two experiments, the results demonstrated that there were faster attention-related responses to dynamic self-faces than to equivalent dynamic changes in the faces of other people. The self-bias effect occurred rapidly after the onset of the stimulus (with a 250 ms SOA), consistent with the self-face being an automatic attractor of attention. In contrast to this, other familiar faces facilitated attentional processing at later stages of information processing (with a 350 ms cue-target SOA); this slower-acting effect may be driven by a top-down expectation from the dynamic changing face cue—even though this was irrelevant for the task and uninformative about the location of the upcoming target.

The ERP results confirmed that self-face cues elicited greater amplitudes in the N1 component over the left central-parietal region, but a smaller amplitude in the P3 component over the right frontal-central-parietal cortex, compared with the face cues of the friend and unfamiliar others. In particular, the magnitude of self-cueing effect (relative to friend) in the N1 component predicted the size of the self-cueing effect in the P3 component. The results indicated that the self-face cueing effect benefits from an enhanced sensory processing to self-faces, which subsequently reduced the probability of uncertainty of the relationship between the cue and its following target (Sutton et al., 1965).

The self-face cueing effect may result from the self-related information automatically gaining attentional salience. Sui, Liu, Mevorach, and Humphreys (2013) had participants learn associations between neutral shapes and labels for the self, friend, and stranger. They subsequently placed the shapes in hierarchical local-global forms and had subjects discriminate between self versus stranger or friend versus stranger shapes at a cues level of the form. Sui et al. found that, irrespective of the level that was cued, there was greater disruption from the self shape as a distractor (to stranger targets) compared with stranger shapes on responses to self targets. This pattern of differential interference did not occur when friend versus stranger discriminations were required. The differential interference effects for self distractors mimic effects found when the perceptual saliency of local and global shapes is manipulated (Mevorach, Humphreys, & Shalev, 2006), when the perceptually salient distractor always differentially disrupts responses to a less-salient target. Here we suggest that self-faces have enhanced attentional salience and consequently attract attention in a rapid fashion. This is consistent with the current study—with the N1 effect reflecting the recruitment of greater attentional resource to the socially salient cue (the self vs. the other faces). Other recent research has also shown that the N1 is modulated by socially-relevant stimuli. For example, relative to when unemotional stimuli are presented, emotional stimuli generate stronger N1 amplitudes (Foti, Hajcak, & Dien, 2009). Here we suggest a similar enhancement through the increased social saliency of self-faces. Whether this links to an inherently greater emotional association to one’s own face compared with other faces (Ma & Han, 2010) is a question for future research. We also found that the magnitude of the self-bias in the N1 predicted P3 activity associated with cue uncertainty.

In Experiment 2, we not only contrasted the participant’s own face with the face of a familiar friend, but also the faces of friends with those of strangers. Here we failed to find any greater N1 activity for the friend-face compared with the face of a complete stranger, and there was no relationship between the N1 and P3 magnitudes for the friend-bias. These last results indicate that the self-bias was not modulated in any simple linear way by familiarity—and indeed there was a lack of evidence for any familiarity effect in the current data. Though the effects of familiarity deserve to be robustly explored, the data suggest that it is the personal relevance of the self-face, rather than its familiarity, that creates the self-bias effect.

Prior work has reported that non-informative eye gazing and body orientation as cues can guide attention and be effective when they are non-predictive of targets (Nummenmaa & Calder, 2009). For example, researchers have reported that non-informative eye gaze as a static central cue (e.g., eyes facing left or right) speeded up responses to subsequent lateralized targets, but only when the SOA before a target was less than 600 ms, but not when there was a longer SOA (1005 ms) (Friesen & Kingstone, 1998). The authors argued that the cueing effect was due to reflexive orienting of attention by participants. Other researchers have found similar effects when head orientation was used as a static cue (Langton & Bruce, 1999) and it has been argued that the gaze effect disappears when there is a longer interval between the cue and target due to participants then responding to the identity rather than gaze orientation of the face (Frischen & Tipper, 2004). Different from past work, the present study used dynamic face cues which mimicked face movements in realistic environments. We found that the dynamic changes in self- and friend-face orientations were both effective for cueing spatial attention in the long SOA condition (350 ms), suggesting that dynamic face cues generate a more sustained attentional response than static gaze cues. In addition, dynamic changes in self-face orientation facilitated spatial attention in the short SOA condition, suggesting early extraction of self-face identity from such images and that identity information can impact on attentional orienting to dynamic faces. The results suggest that the presence of dynamic changes in face cues may be particularly important for attentional orienting and can combine with, rather than compete with, face identity for attracting attention.

Overall, the results indicate that the dynamically changing image of your own face acts as an automatic attractor of attention. Through this, self-faces will gain more attentional resource for sensory processing and subsequently affect decision-making by reducing the probability of uncertainty in the relation of the cue to the target. The results are consistent with the notion that the self-face is a particularly important ecological stimulus for attention.

## Notes

No potential conflict of interest was reported by the authors.

## References

[CIT0001] Axelrod V., Bar M., Rees G. (2015). Exploring the unconscious using faces. *Trends in Cognitive Sciences*.

[CIT0002] Brédart S., Delchambre M., Laureys S. (2006). Short article one’s own face is hard to ignore. *The Quarterly Journal of Experimental Psychology*.

[CIT0003] [Computer software]. http://www.pstnet.com/eprime.cfm.

[CIT0004] Foti D., Hajcak G., Dien J. (2009). Differentiating neural responses to emotional pictures: Evidence from temporal-spatial PCA. *Psychophysiology*.

[CIT0005] Friesen C. K., Kingstone A. (1998). The eyes have it! Reflexive orienting is triggered by nonpredictive gaze. *Psychonomic Bulletin & Review*.

[CIT0006] Frischen A., Tipper S. P. (2004). Orienting attention via observed gaze shift evokes longer term inhibitory effects: Implications for social interactions, attention, and memory. *Journal of Experimental Psychology: General*.

[CIT0007] Gronau N., Cohen A., Ben-Shakhar G. (2003). Dissociations of personally-significant and task-relevant distractors inside and outside the focus of attention: A combined behavioural and psychophysiological study. *Journal of Experimental Psychology: General*.

[CIT0008] Haider M., Spong P., Lindsley D. B. (1964). Attention, vigilance, and cortical evoked-potentials in humans. *Science*.

[CIT0009] Keyes H., Dlugokencka A. (2014). Do I have my attention? Speed of processing advantages for the self-face are not driven by automatic attention capture. *Plos One*.

[CIT0010] Langton S. R. H., Bruce V. (1999). Reflexive visual orienting in response to the social attention of others. *Visual Cognition*.

[CIT0011] Luck S. J., Woodman G. F., Vogel E. K. (2000). Event-related potential studies of attention. *Trends in Cognitive Sciences*.

[CIT0012] Ma Y., Han S. (2010). Why we respond faster to the self than to others? An implicit positive association theory of self-advantage during implicit face recognition. *Journal of Experimental Psychology: Human Perception and Performance*.

[CIT0013] Mevorach C., Humphreys G. W., Shalev L. (2006). Opposite biases in salience-based selection for the left and right posterior parietal cortex. *Nature Neuroscience*.

[CIT0014] Nummenmaa L., Calder A. J. (2009). Neural mechanisms of social attention. *Trends in Cognitive Science*.

[CIT0015] Ro T., Russell C., Lavie N. (2001). Changing faces: A detection advantage in the flicker paradigm. *Psychological Science*.

[CIT0016] Sui J., Han S. (2007). Self-construal priming modulates neural substrates of self-awareness. *Psychological Science*.

[CIT0017] Sui J., Liu C. H., Han S. (2009). Cultural difference in neural mechanisms of self-recognition. *Social Neuroscience*.

[CIT0018] Sui J., Liu M., Mevorach C., Humphreys G. W. (2013). The salient self: The left intra-parietal sulcus responds to social as well as perceptual-salience after self-association. *Cerebral Cortex*.

[CIT0019] Sutton S., Braren M., Zubin J., John E. R. (1965). Evoked-potential correlates of stimulus uncertainty. *Science*.

[CIT0020] Tao R., Zhang S., Li Q., Geng H. (2012). Modulation of self-esteem in self-and other-evaluations primed by subliminal and supraliminal faces. *Plos One*.

[CIT0021] Tong F., Nakayama K. (1999). Robust representations for faces: Evidence from visual search. *Journal of Experimental Psychology: Human, Perception and Performance*.

